# Quantitative Analysis of *Panax ginseng* by FT-NIR Spectroscopy

**DOI:** 10.1155/2014/741571

**Published:** 2014-05-05

**Authors:** Xin-fang Xu, Li-xing Nie, Li-li Pan, Bian Hao, Shao-xiong Yuan, Rui-chao Lin, Hai-bo Bu, Dan Wang, Ling Dong, Xiang-ri Li

**Affiliations:** ^1^School of Chinese Materia Medica, Beijing University of Chinese Medicine, No. 6 Wangjing Zhonghuannan Road, Beijing 100102, China; ^2^National Institutes for Food and Drug Control, State Food and Drug Administration, No. 2 Tiantan Xili, Beijing 100050, China

## Abstract

Near-infrared spectroscopy (NIRS), a rapid and efficient tool, was used to determine the total amount of nine ginsenosides in *Panax ginseng*. In the study, the regression models were established using multivariate regression methods with the results from conventional chemical analytical methods as reference values. The multivariate regression methods, partial least squares regression (PLSR) and principal component regression (PCR), were discussed and the PLSR was more suitable. Multiplicative scatter correction (MSC), second derivative, and Savitzky-Golay smoothing were utilized together for the spectral preprocessing. When evaluating the final model, factors such as correlation coefficient (*R* ^2^) and the root mean square error of prediction (RMSEP) were considered. The final optimal results of PLSR model showed that root mean square error of prediction (RMSEP) and correlation coefficients (*R* ^2^) in the calibration set were 0.159 and 0.963, respectively. The results demonstrated that the NIRS as a new method can be applied to the quality control of *Ginseng Radix et Rhizoma*.

## 1. Introduction

Ginseng, the dried root and rhizome of* Panax ginseng* C.A. Meyer, [1] is one of the most famous and valuable traditional Chinese herbs in Asia. In recent years, it has been widely used for making functional foods in many other countries like in Europe and in the USA. Ginsenosides are confirmed to be the principal active compounds of ginseng and its products. Ginsenosides are named as “Rx,” where the “R” stands for the root and the “x” describes the chromatographic polarity in an alphabetical order [2]. According to different aglycones, ginsenosides can be classified into three types: the 20(S)-protopanaxadiol type such as ginsenoside Rb_1_, Rc, Rb_2_, and Rd, the 20(S)-protopanaxatriol type such as ginsenoside Rg_1_ and Re, and the oleanolic acid type including ginsenoside Ro and polyacetyleneginsenoside Ro, respectively. There are compositional differences between the types of ginseng with respect to the 7 fingerprint ginsenosides (Rb_1_, Rb_2_, Rc, Rd, Re, Rg_1_, and Rf) that are often measured to standardize ginseng extracts. With the exception of Rf, the 6 ginsenosides are the most abundant ginsenosides in the* Panax ginseng* [3]. Even the rare ginsenosides such as ginsenoside Rb_3_, Rf, and Rg_2_ also have significant pharmacological activities [4]. Ginsenosides have many pharmaceutical effects, for example, an anticarcinogenic effect, an immune-modulatory effect, anti-inflammatory, antiallergic effects, and so on [5]. In general, ginseng and even a single compound of ginsenoside produce their effects on multiple sites of action, which makes it an ideal candidate to develop multitarget drugs. Recently, ginsenosides have been found to play an important role in the central nervous system. These diseases are prevalent all over the world, which include Alzheimer's disease, Parkinson's disease, cerebral ischemia, depression, and many other neurological disorders including neurodevelopmental disorders [6].

The analysis of ginsenosides has been performed with various analytical methods such as thin layer chromatography (TLC) [7], GC [8], high performance liquid chromatography (HPLC) [9], capillary electrophoresis [10], tandem instrumentation of high performance liquid chromatography with mass spectroscopy (HPLC-MS) [11], and enzyme immunoassay [12], while the high performance liquid chromatography has been routinely used. But all these methods need complex preliminary treatments of the samples; meanwhile they also spend a lot of time and energy. Additionally, many methods of quantitative analysis must have the standards of ginsenosides as reference.

By contrast, the near-infrared spectrometry (NIRS) technology is an appropriate alternative method. NIRS has been widely applied in various areas, such as foods, pharmaceuticals, and petroleum [13, 14]. The advantages of this technique are mainly attributed to its speed, economy, accuracy, and precision in comparison with other analytical techniques. Recently, NIRS has been widely employed in the study of traditional Chinese herbs for qualitative analysis [15] and quantitative analysis [16]. Liu et al. [17] have developed a method for the determination of ginsenoside Rb_1_, Re, and Rb_1_, as well as the total ginsenosides in folium ginseng by near-infrared spectroscopy.

In the study, the total amount of nine different ginsenosides (ginsenoside Rb_1_, Rb_2_, Rb_3_, Rc, Rd, Re, Rf, Rg_1_, and Rg_2_) in* Panax ginseng* was determined. A new analytical method was developed using FT-NIR with multivariate regression methods. This is the first report for the quantitative analysis of the total amount of nine ginsenosides in ginseng by NIRS.

## 2. Experiment Methods

### 2.1. Sample Preparation

56 ginseng samples, in which 29 samples were from Fusong County of Jilin province of China, 20 samples from Ji'an City of Jilin province, and 7 samples from Tonghua County of Jilin province, were collected for the content determination. These 56 samples were pulverized into powder and sieved on a sieve of 65 meshes. To ensure the moisture did not interfere as far as possible, these 56 samples were heated for 6 h at 50°C in a dryer.

All of these samples were identified by Professor Xiangri Li (School of Chinese Materia Medica, Beijing University of Chinese Medicine) and deposited in the specimen cabinet of traditional Chinese medicine of Beijing University of Chinese Medicine.

### 2.2. NIR Spectra Collection

Near-infrared diffuse reflectance spectra of ginseng samples were acquired using an NIR system (Thermo Electron Corp., USA) with an Integrating Sphere Module over the wavenumber range of 10000–4000 cm^−1^ and recorded in absorbance with air as the reference standard. The spectra were collected at the resolution of 8 cm^−1^ and the interval of 2 cm^−1^ per spectrum by averaging 64 scans. The InGaAs detector and the software of RESULT 3.0 were used to collect the NIR spectra.

### 2.3. Determination of Nine Kinds of Ginsenosides

**Figure 1:**
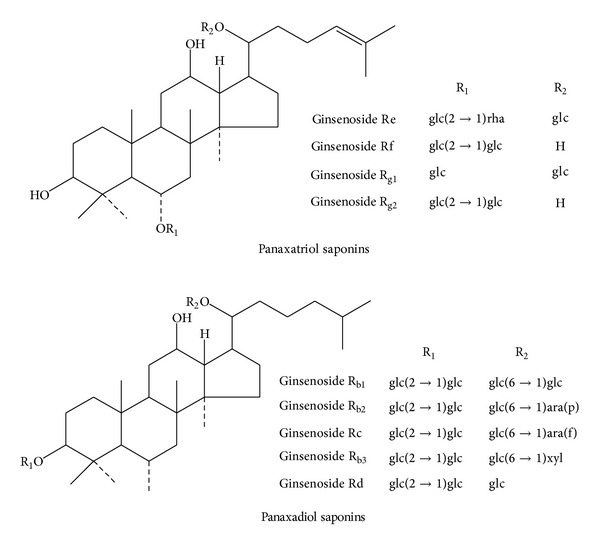
Chemical structures of the nine ginsenosides.

The chemical structures of the nine ginsenosides are shown in Figure 1. We developed a method for simultaneous determination of nine kinds of ginsenosides [18].  Table 1 lists the linear regression equations of these nine kinds of ginsenosides.

For each sample, 0.4 g of powder, precisely weighed, was extracted by refluxing twice with 50 mL of methanol for 60 min each time. After cooling, both extracts were evaporated to dryness and then dissolved in and diluted to the volume scale by methanol in a 10 mL volumetric flask. This solution was filtered through a 0.45 *μ*m membrane filter and injected into the HPLC system.

A Waters 1525 HPLC system consisting of four pumps, on line degasser, a thermostat maintained at 30°C, and a Waters 2487 UV detector was used in this study. The chromatographic separation was accomplished on an Agilent Zorbax column SB-C18 (250 × 4.6 mm, 5 *μ*m). The mobile phase consisted of (A) H_2_O and (B) acetonitrile (v/v). The linear gradient program was shown in Table 2. The flow rate was set at 1.0 mL/min and the sample injection volume was 10 *μ*L. The absorbance was measured at a wavelength of 203 nm.

### 2.4. Spectral Data Preprocessing

While the spectral data was acquired by the NIR instrument, the noise, baseline drift, and scatter effects have simultaneously appeared in the spectra. It is indispensable to preprocess the spectra before modeling. Multiplicative scatter correction (MSC) [19] was developed to eliminate the scatter effects caused by different particle size distribution. The processing by spectral derivatives [20] can protect against the influence of baseline drift. It was effective to discriminate overlapping peaks and increase the resolution and sensitivity. To avoid enhancing the noise induced by the derivatives, the spectra need to be smoothed. The frequently used smoothing methods are the Savitzky-Golay (SG) filter [21] and the Norris derivative (ND) filter.

### 2.5. Multiple Multivariate Regression Methods and Software

Partial least square regression (PLSR) and principal component regression (PCR) were used to establish the quantitative models in the study [22]. The performance of the final model was evaluated in terms of RMSEC, RMSEP, and R^2^. The models in this study were all conducted with the chemometric software TQ analyst 7.2 (Thermo Electron Corp., USA).

## 3. Results and Discussion

### 3.1. Results and Analysis of Content Determination

The results which determined by HPLC were regarded as actual values (or called reference values) to establish the calibration models. Before the modeling of NIR, the Chauvenet test [23] was applied to find the spectral outliers at the 90% confidence level using the TQ software. The spectra that failed the Chauvenet test were judged as spectral outliers and neglected firstly. After the test, 4 spectral outliers were removed from the ginsenosides model.

The remaining 52 samples were divided into the calibration set and the validation set with the ratio of 3 : 1. For each model, the calibration set was used to develop the calibration model and the validation set was used to predict the calibration model. This can make sure that the model was stable and precise. It must be guaranteed that the ranges of the actual values in the validation set cover the values in validation set (see Table 3). The best calibration equation for each analysis was selected in terms of the lowest root mean square error of cross-validation (RMSECV), root mean square error of calibration (RMSEC), root mean square error of prediction (RMSEP), and the highest correlation coefficient (*R* ^2^).

### 3.2. NIR Calibration Model

#### 3.2.1. Spectral Regions Choosing

**Figure 2:**
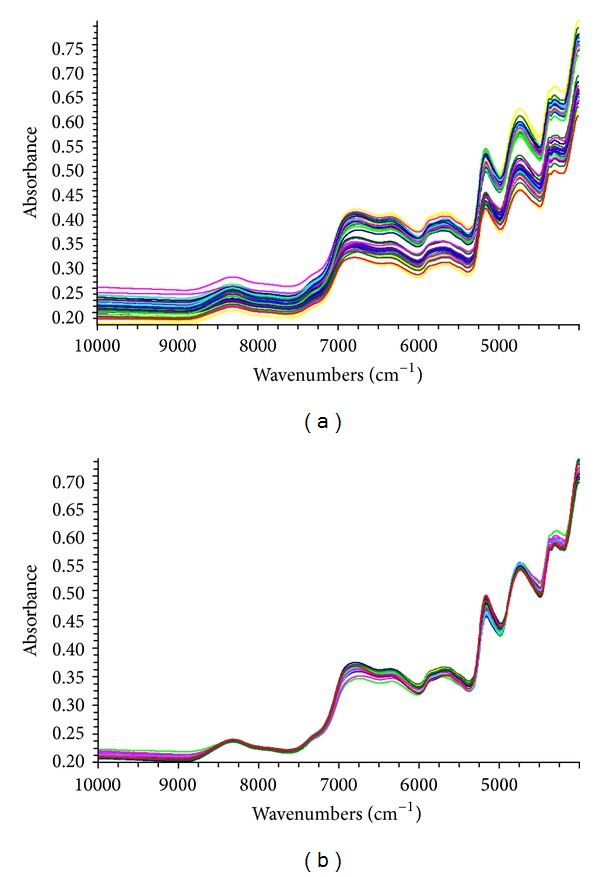
NIR spectra of ginseng samples obtained from original data (a) and MSC processing (b).

NIR spectra of samples are shown in Figure 2(a). It can be easily found that the spectral regions, 10000–9000 cm^−1^ and 4100–4000 cm^−1^, present a high noise level. When choosing the modeling regions, the above spectral absorption information must not be neglected. After many trials, 8367.18–4242.6 cm^−1^ for ginsenosides was chosen to establish the calibration models.

#### 3.2.2. Spectral Preprocessing

**Figure 3:**
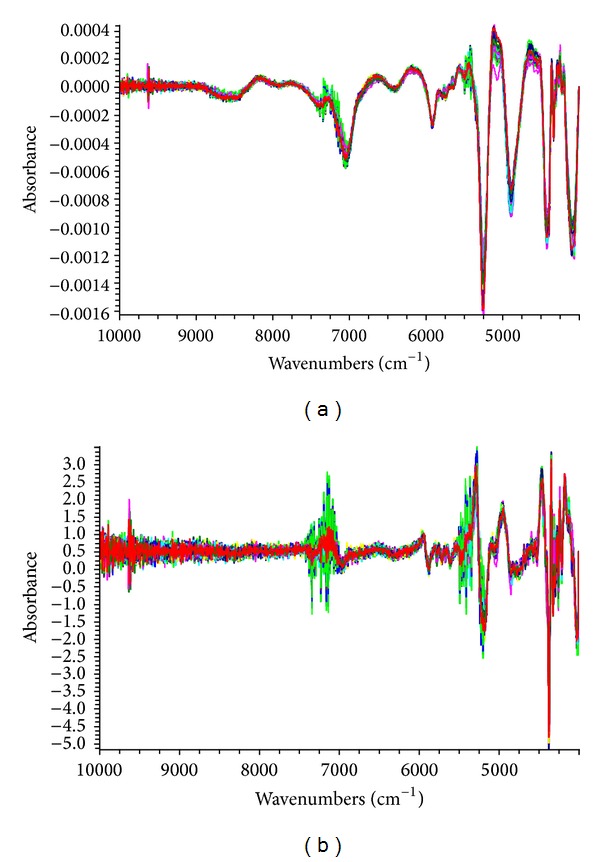
NIR spectra for ginseng samples processed by 1st derivative + SG + MSC (a) and MSC + SG + 2nd derivative (b).

Figure 2 shows the spectra acquired from original data and MSC processing. The great change is that the differences of all the NIR spectra get much smaller after MSC processing. The spectra preprocessing was chosen (Table 4) by the lowest RMSEP and RMSEC and the highest* R* ^2^. The optimum spectra processing was MSC + SG + 2nd derivative and was used for the total amount of nine ginsenosides. It also can be observed that the main absorption peaks are distributed in the selected region from Figure 3.

#### 3.2.3. Effect of Statistical Model

Table 5 shows the model results with the different statistical algorithms. PLSR was superior to PCR for developing the calibration model for the total amount of nine ginsenosides between the two statistical models.

#### 3.2.4. Optimization of Factors of Models

Each calibration model has an optimum number of factors. The appropriate number of factors can be used for preventing the occurrence of underfitting and overfitting. The optimum numbers of factors were chosen according to which corresponded to the lowest RMSECV obtained by leave-one-out cross-validation (LOOCV) [24]. The optimum numbers of factors were 4 in the model of ginsenosides.

#### 3.2.5. Best Calibration Models

**Figure 4:**
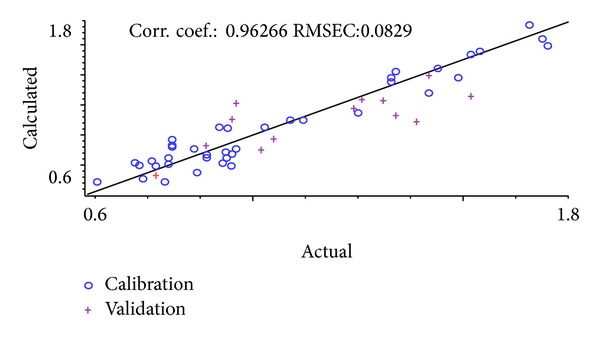
Correlation diagram between the NIR model calculated values and the actual values of the total amount of nine ginsenosides.

Usually, RMSEC, RMSEP, and corresponding* R* ^2^ values are used for evaluating the NIR calibration models. Table 5 lists the RMSEC, RMSEP, and* R* ^2^ data for the optimum calibration equations of the total amount of nine ginsenosides using the above optimum parameters. Correlation diagram between the NIR model calculated values and the actual values is shown in Figure 4. It can be observed that the correlation coefficient of calibration model is 0.963, which shows a good correlation between the actual values and the NIR model calculated values. Additionally it is also suggested that the stable and precise model established can be used for quantitative analysis of the chemical compositions in* Ginseng Radix et Rhizoma*.

## 4. Conclusions

A method for determining the total amount of nine ginsenosides in* Panax ginseng* was developed using FT-NIR with multivariate regression methods for the first time. The results indicate that the model is precise and stable and has the good properties of prediction. Compared with traditional methods such as HPLC and TLC, NIRS has great merits for nondestruction, convenience, and environmental protection. Ginsenosides in* Panax ginseng* can be analyzed quickly and easily from the study. Additionally, it provides a new idea for the quality control of* Panax ginseng* in many commodities.

## Figures and Tables

**Table 1:** The linear regression equations of nine ginsenosides.

Ginsenoside	Regression equation	Linear ranges/*μ*g	*R* ^2^
Rg_1_	*Y *= 383524*X *− 65359	0.456–2.280	0.9997
Rb_1_	*Y *= 3080694*X *− 2960	0.516–2.580	0.9999
Re	*Y* = 375112*X *− 76321	0.382–1.910	0.9994
Rf	*Y *= 407560*X −* 43377	0.174–0.870	0.9999
Rc	*Y *= 290956*X* − 62647	0.342–1.710	0.9999
Rb_2_	*Y *= 288739*X* − 46577	0.253–1.270	0.9998
Rg_2_	*Y =* 469440*X* − 23253	0.034–0.170	0.9998
Rb_3_	*Y *= 419332*X *− 14938	0.029–0.150	0.9998
Rd	*Y *= 650313*X* − 24730	0.068–0.340	0.9993

**Table 2:** The linear gradient program of nine kinds of ginsenosides.

	Time (min)								
	0	30	45	60	90	100	105	110	120
H_2_O-A (%)	81	81	72	72	65	60	5	81	81
Acetonitrile-B (%)	19	19	28	28	35	40	95	19	19

**Table 3:** The content ranges of ginsenosides from calibration and validation set.

	Sets	Number of samples	Rang (%)	Mean (%)
Ginsenosides	Calibration set	39	0.63–1.70	0.83
	Validation set	13	1.42–1.70	1.49

**Table 4:** Optimization of spectra processing in carlibration models.

Preprocessing	RMSEP	RMSEC	*R* ^2^
MSC + SG	0.254	0.302	0.656
MSC + SG + 1st derivative	0.266	0.186	0.886
MSC + SG + 2nd derivative	0.159	0.083	0.963
MSC + ND + 1st derivative	0.252	0.269	0.739
MSC + ND + 2nd derivative	0.403	0.124	0.852

**Table 5:** RMSEC, RMSEP, and *R* ^2^ for the calibration models.

Algorithm	RMSEP (%)	RMSEC (%)	*R* ^2^
PLSR	0.159	0.083	0.963
PCR	0.360	0.233	0.673
